# Biological Pathways and Potential Targets for Prevention and Therapy of Chronic Allograft Nephropathy

**DOI:** 10.1155/2014/482438

**Published:** 2014-05-27

**Authors:** Badri Man Shrestha, John Haylor

**Affiliations:** Division of Renal Transplantation, Sheffield Kidney Institute, Northern General Hospital, Herries Road, Sheffield S5 7AU, UK

## Abstract

Renal transplantation (RT) is the best option for patients with end-stage renal disease, but the half-life is limited to a decade due to progressive deterioration of renal function and transplant failure from chronic allograft nephropathy (CAN), which is the leading cause of transplant loss. Extensive research has been done to understand the pathogenesis, the biological pathways of fibrogenesis, and potential therapeutic targets for the prevention and treatment of CAN. Despite the advancements in the immunosuppressive agents and patient care, CAN continues to remain an unresolved problem in renal transplantation. The aim of this paper is to undertake a comprehensive review of the literature on the pathogenesis, biological pathways of RT fibrogenesis, and potential therapeutic targets for the prevention and therapy of CAN.

## 1. Introduction

Renal transplantation (RT) is the best form of treatment for end-stage renal disease (ESRD), because this improves the quality of life and patient survival and is cost-effective [1]. Chronic allograft nephropathy (CAN) is the leading cause of late RT loss; hence, attention has been focussed on understanding the pathogenesis of RT fibrogenesis and interventional strategies to prevent and treat CAN [2].

CAN is characterised by a relatively slow but variable rate of decline in renal function after first 3 months of RT, often in combination with proteinuria and hypertension [3]. CAN should be differentiated from other causes of transplant dysfunction such as rejection (acute, subclinical, and chronic), calcineurin inhibitor (CNI) nephrotoxicity, glomerulonephritis (recurrent and de novo), nephrosclerosis (secondary to old donor age, recipient hypertension, hyperlipidaemia, and smoking), and others (ureteric obstruction, BK virus nephropathy, and transplant renal artery stenosis) [4, 5].

Schweitzer et al. from Minnesota in 1991 reported, in a cohort of 2396 patients over a period of 20 years (1970–1989), chronic rejection as the leading cause of graft loss following RT amounting to 24%, followed by death with functioning graft (18%), infection (13%), and acute rejection (11%) [6]. More lately, Sijpkens et al. from Netherlands reported that 54 of the 654 (8%) RTs performed between 1983 and 1997 had histological evidence of CAN and CAN accounted for 37% of graft loss after first 6 months post-RT [7]. Naesens et al. have reported that the global burden of early chronic histological damage within the first year after transplantation significantly affected the long-term survival of the allografts [8]. Currently, chronic antibody-mediated rejection from both anti-human leucocyte antigen (HLA) antibodies and non-HLA antibodies is being recognised as an important cause of CAN [9, 10].

The aim of this review is to consolidate the published evidence on the pathogenesis, biological pathways of RT fibrogenesis, and potential therapeutic targets for the prevention and therapy of CAN.

## 2. Literature Search Strategy

The literature search was carried out in PubMed and relevant websites using the words “renal transplantation,” “chronic allograft nephropathy,” “chronic rejection,” “graft loss,” “transplant fibrosis,” and “prevention,” Relevant references were compiled in the EndNote software (X6.0.1; Bld 6599).

## 3. Risk Factors Implicated in CAN

Both antigen-dependent (immunological) and antigen-independent (nonimmunological) factors are implicated in the aetiology of CAN (Table 1). On occasions, it is difficult to pinpoint a single aetiological factor, as more than one factor is usually implicated in the pathogenesis of CAN [11]. Recurrent episodes of acute tubular-interstitial rejection can explain the interstitial fibrosis and tubular atrophy observed in some cases. Cytokines released during episodes of rejection, including interleukin-1, fibroblast growth factor, and platelet derived growth factor, are likely to play a role in promoting the fibroblast and smooth muscle proliferation seen in allograft vessels. In cases with prior documented intimal arteritis, vessel thickening can be explained as a direct result of immunologic vascular injury. Graft atherosclerosis leads to ischaemic glomerulopathy [12].

Once glomerulosclerosis has developed, the remaining glomeruli undergo compensatory hypertrophy, increased glomerular capillary hydraulic pressure, and increased glomerular filtration. These haemodynamic forces damage the glomerular capillary endothelium, cause mesangial expansion, and accentuate the evolution of chronic transplant glomerulopathy [12]. In support of this hypothesis, it has been shown experimentally that if the increase in glomerular filtration rate is prevented by putting animals on a severely protein restricted diet, the rate of progression of glomerular sclerosis in allograft kidneys is retarded [13, 14]. Arteriolosclerosis and interstitial fibrosis in the allograft may also occur as a result of hypertension, recurrent pyelonephritis, and chronic cyclosporine or tacrolimus toxicity. The relative contribution of these various processes to the ultimate loss of any given allograft may be difficult to determine by pathological evaluation alone. The aetiologically noncommittal term “chronic allograft nephropathy” was in fact coined to accommodate this difficulty [15].

## 4. Pathology of CAN

**Figure 1:**
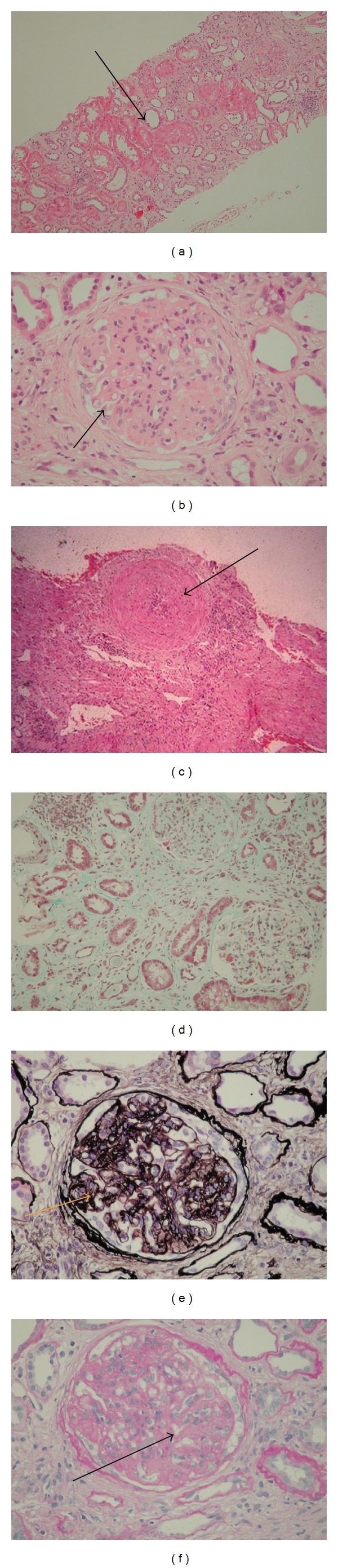
Haematoxylin and eosin (H & E) stain showing (a) tubular atrophy, interstitial fibrosis (→), (b) glomerulosclerosis (→), and (c) concentric obliterative arteriolopathy (→); (d) Masson's trichrome stain showing interstitial fibrosis (→); (e) silver stain (→); and (f) PAS stain showing double contour of glomerular capillary basement membrane (→) (magnification ×20) (source: Northern General Hospital, Sheffield).

The kidney affected by CAN looks pale and fibrotic with a dense, thickened, adherent capsule. Under* light microscopy*, characteristic changes are found in the glomerular, tubule-interstitial, and microvascular compartments (Figure 1).

### 4.1. Microvascular Changes

The “transplant arteriopathy” characterised by severe intimal proliferation and luminal narrowing associated with sparse infiltration of T-cells and macrophages is seen in all arteries extending from main renal artery to the interlobar arteries. The intima shows concentric fibrous thickening with intact internal elastic lamina. The matrix appears pale in haematoxylin eosin-stained sections and contains acid mucopolysaccharides, collagen, and increased hyaluronic acid. The cells in the intima include *α*-smooth actin-positive myofibroblasts and smooth muscle cells. The media generally shows no abnormality. Arterioles do not show intimal changes. Hyalinosis of arteries is a feature of CNI toxicity, never seen in animal models not on CNI drug treatment [16].

### 4.2. Glomerular Changes

In CAN, ischaemic glomeruli, atubular glomeruli, and chronic transplant glomerulopathy are the histological findings. Morphometric analysis of CAN shows populations of small (ischaemic) and large (hyperfiltrating) glomeruli. The ischaemic glomeruli are characterised by wrinkling and collapse of glomerular capillary wall associated with extracapillary fibrotic material [17].

Chronic transplant glomerulopathy (CTG) comprises a spectrum of histological abnormalities which include thickening or duplication of glomerular capillary basement membrane (double contour lesion) and mesangial expansion. CTG implies chronic endothelial injury of glomerular capillary loops and is clinically accompanied by substantial or nephrotic range proteinuria, renal function impairment, and reduced RT survival [18].

### 4.3. Tubulointerstitial Changes

As a result of ischaemia caused by microvascular changes described above, the tubules undergo atrophy, which may also result from tubulitis. The tubular basement membranes (TBM) are thickened and duplicated, but some show pronounced shrinkage and thinning of TBM and thereby dilatation. The interstitium shows fibrosis with variable mononuclear infiltrate with small lymphocytes, plasma cells, and mast cells. The fibrosis can have different patterns, such as dense and focal, diffuse and fine, striped, or subcapsular. The peritubular capillaries (PTC) are depleted leaving behind only traces of original basement membrane as seen on silver- or PAS-stained sections [19].

### 4.4. Electron Microscopy

**Figure 2:**
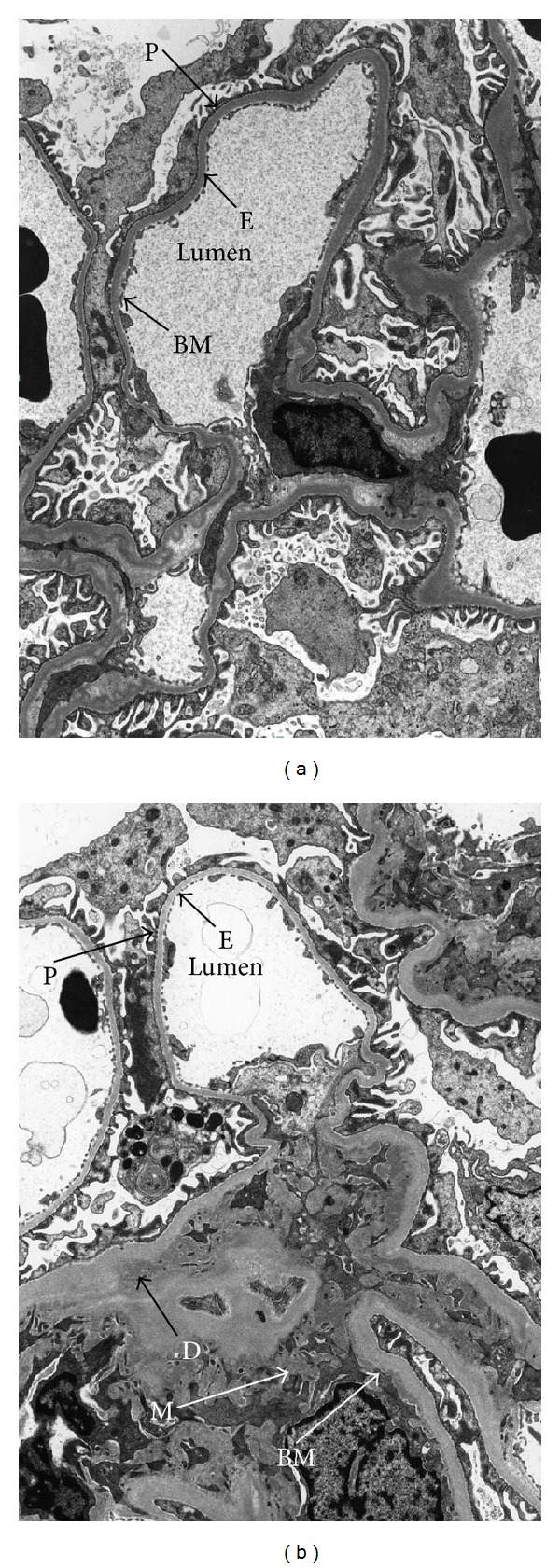
Electron microscopy showing (a) a normal glomerulus (E: endothelial cell; P: podocytes; Lumen: capillary lumen); and (b) transplant glomerulopathy with presence of a well-developed basement membrane (BM) along the entire capillary circumference, mesangial expansion (M), and accumulation of subendothelial deposit (D) (magnification ×7000) (source: Northern General Hospital, Sheffield).

Under electron microscopy, the CTG is associated with deposition of flocculent or fibrillary material; mesangial cellular proliferation with matrix expansion; multilamination or multilayering of the capillary basement membrane. Multilamination of capillary basement membrane as high as seven or more layers indicates past or recent endothelial injury with subsequent repair, which was present in 38% of failed transplants ascribed to CAN (Figure 2) [20].

## 5. Theories of Pathogenesis of CAN

CAN represents the summated effects of tissue injury resulting from several pathogenic insults and the healing response of the kidney to injury, in addition to the influence of alloimmunity and immunosuppression. Various hypotheses have been proposed to explain the pathogenesis of CAN, which include (1) chronic rejection, (2) input-stress model, (3) cumulative damage, (4) oxidative stress, (5) cytokine excess, (6) epithelial-mesenchymal transition (EMT) induced fibrosis, (7) replicative senescence, (8) insertion/deletion polymorphism of angiotensin-converting enzyme, and (9) acetylcholine excess [21–30].

## 6. Biological Pathways in Allograft Fibrosis

Majority of extracellular matrix (ECM) in the kidney is composed of collagens I, III, V, VII, and XV and fibronectin. The structural framework is formed by proteoglycans, polysaccharides, and glycoproteins. The tubular basement membrane consists of collagen IV and laminin. Fibrosis or scarring is the overgrowth and sclerosis of the tissues due to the excessive deposition of ECM, which becomes pathological when the functioning architecture is destroyed, leading to functional impairment [31].

The pathway of progression of fibrosis in RT kidney leading to RT loss comprises time-dependent series of pathological insults causing histological changes. There are two broad phases of RT damage observed in sequential biopsy studies, starting with* early* tubulointerstitial injury [32] followed by* later *microvascular and glomerular abnormalities and further progressive interstitial fibrosis and tubular atrophy [23].

**Figure 3:**
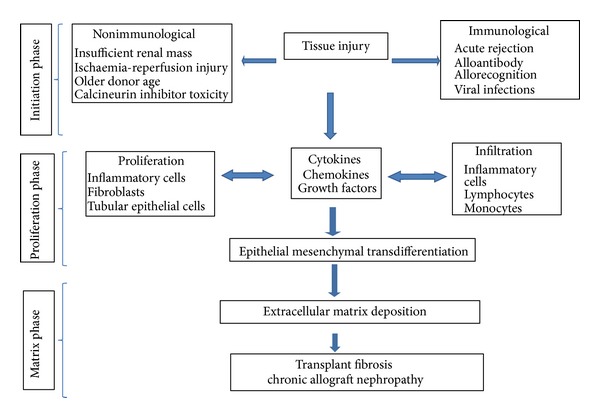
Showing pathways of fibrosis in chronic allograft nephropathy.

Figure 3 summarises the events leading fibrosis to CAN and can be arbitrarily divided into three phases, namely, the* initiation phase, *the* fibrogenesis phase, *and the* matrix accumulation phase.* In the initiation phase, tissue injury is caused by antigen-dependent of antigen-independent factors. Regardless of the nature of initiating agent, fibrogenesis phase sets in, which consists of inflammatory and proliferative responses regulated by chemokines, cytokines and growth factors. The cascade of events results in the matrix accumulation phase due to either increased production and/or decreased degradation of matrix, culminating in fibrosis [33].

Injury to the vascular endothelium, glomerular, or tubular epithelium caused by antigen-dependent (immunological) or antigen-independent pathways (toxic, ischaemic, or mechanical) leads to the secretion of proinflammatory mediators (lipid mediators, chemokines, cytokines, adhesion molecules, and growth factors) by all intrinsic renal cells. Expression of adhesion molecules and chemokines by the endothelial cells of the glomerular and peritubular capillaries supports leucocyte arrest and transmigration either into the mesangium or the interstitial space. The infiltration and proliferation of leucocytes enhances the local production of cytokines and chemokines. Furthermore, the neutrophils and macrophages generate reactive oxygen species and lipid mediators contributing to local tissue damage and glomerular and tubulointerstitial inflammation, which results in haematuria, leukocyturia, and proteinuria [34, 35].

Macrophages themselves secrete ECM components, but they are also major source of growth factors such as fibroblast growth factor (FGF), transforming growth factor-*β* (TGF-*β*), tumour necrosis factor-*α* (TNF-*α*), epithelial growth factor (EGF), and platelet-derived growth factor (PDGF). The TGF-*β* superfamily includes three TGF-*β* isoforms (TGF-*β*1, TGF-*β*-2, and TGF-*β*-3), activins, and bone morphogenetic proteins (BMPs) [36]. Both antigen-dependent and independent injuries lead to the expression of TGF-*β* isoforms, which act by engaging intracellular signalling cascades of canonical SMAD or noncanonical, non-SMAD family of proteins. The SMAD pathways are activated/phosphorylated by TGF-*β*1 receptors such as activins-receptor like kinase (ALK) 5 and ALK1. However, TGF-*β* also activates the noncanonical, SMAD-independent pathways such as Ras/rho and MAPK, NF-kB, or PI3kinase/AKT pathway. Receptor-activated SMAD protein complexes translocate within the nucleus and initiate transcription of target genes [37–39].

TGF-*β*1 is the key modulator of glomerulosclerosis, tubulointerstitial fibrosis, and EMT in the kidney. TGF-*β*1 sets off a cascade of profibrotic molecules through the activation of SMAD2/3 [40] and MEK signalling pathways [41], which results in the transcription of genes and activation of molecules involved in matrix deposition and fibrosis. Proinflammatory and profibrotic cytokines stimulate the proliferation of mesangial cells in the glomerulus and of interstitial fibroblasts in the interstitium, which, upon activation, increase the synthesis of extracellular matrix components. Leucocyte infiltration, fibroblast proliferation, and matrix deposition as well as oedema increase the interstitial volume. There is proliferation of resident fibroblasts and of the myofibroblasts derived from tubular epithelial cells by a process of EMT, induced by the macrophage-derived, profibrotic cytokine FGF-2 [42]. In the glomeruli, stimulated mesangial cells secrete the collagen type IV, laminin, and fibronectin that contribute to glomerulosclerosis [43]. Mesangial expansion leads to narrowing, obliteration, or dilatation of glomerular capillaries. This results in damage to podocytes and downstream peritubular capillaries leading to destruction of entire nephrons [44].

The tubulointerstitial compartment shows progressive expansion of ECM through continuous stimulation of fibroblasts. Transdifferentiation of activated tubular epithelial cells leads ECM expansion. Massive increase in interstitial volume from infiltrates and expansion of ECM leads to an increase in the distance between the peritubular capillaries and the tubules, thereby impairing oxygen diffusion as well as tubular reabsorption and excretory function [45]. The tubulointerstitial ischaemia is considered to be an important factor for tubular cell apoptosis, necrosis, and tubular atrophy [46]. Finally, extensive loss of transplant renal parenchyma leads to transplant failure. At a late stage of CAN, although the leucocytic infiltration resolves, renal fibroblasts maintain the synthesis of ECM due to sustained hypoxia and autocrine stimulation [47]. Myofibroblasts contribute to the contraction of the fibrous tissue with scarring leading to a shrunken transplant kidney at the terminal stage.

## 7. Potential Targets for Prevention and Therapy of CAN

The modulation of fibrosis is central in RT. As CAN is the end result of injury caused by acute rejection, infection, ischaemia reperfusion injury, alloantibody-mediated rejection, and drug toxicity, each factor needs to be prevented and treated appropriately. Shortening of cold ischaemia time, HLA-matching and reduced sensitisation, avoidance of CNI-toxicity, cytomegalovirus prophylaxis, and treatment of subclinical rejection detected through protocol biopsies are paramount [48]. Early detection of changes of CAN by microarray mRNA assays, which detect alteration in the transcriptomes at a very early stage of interstitial fibrosis, even before infiltration by inflammatory cells, has opened up a new avenue for interventions at this stage [35, 49]. At the molecular level, targeting the blockade of synthesis or action of enzymes, cytokines, chemokines, and growth factors at various stages of renal fibrogenesis is being investigated, both in experimental and clinical settings, which are discussed below.

### 7.1. Chemokine Blockade

Blockade of chemokines (BX471: CCR1 antagonist; and met-RANTES: RANTES antagonist) is proven to be effective in preventing RT fibrosis in rat transplant models. No parallel study has been carried out in human RTs [50, 51]. Chemokines are a family of small size (8–10 kDa) chemotactic cytokines, which, in transplantation, induce recruitment and activation of T cells and monocytes or macrophages. So far more than 50 chemokines and 20 chemokine receptors have been identified. Members of chemokines family are divided into four groups (C, CC, CXC, and CX_3_C) based upon the position of their first two cysteine residues. They are further classified as inflammatory or haemostatic.

In Fisher-to-Lewis rat allografts, BX-471, chemokine receptor type 1 (CCR1) antagonist, prevented the infiltration of T cells and macrophages, decreased cell proliferation (ED1, CD8, and Ki67), and reduced expression of acute phase reactive proinflammatory genes (HO-1, osteopontin) and molecules associated with fibrosis (PAI-1, TGF-*β*, and biglycan). There was a significantly lower number of SMA-positive interstitial myofibroblasts and reduction in the deposition of collagen [51].

Similarly, Met-RANTES, a chemokines receptor antagonist (CCR5), in Fisher-to-Lewis allografts, blocked the effects of RANTES (regulated on activation, normal T-cell expressed) reducing the infiltration of lymphocytes and macrophages in allografts, accompanied by decreased mRNA expression of interleukin (IL)-2, IL-1 beta, tumour necrosis factor-alpha (TNF-alpha), and RANTES and thereby reduced glomerulosclerosis, tubulointerstitial fibrosis, and proteinuria [50].

### 7.2. Blockade of Oxidative Stress

In human RT, intraoperativeadministration of recombinant human superoxide dismutase decreased the incidence of acute and chronic rejection [52], whereas treatment pre- and postreperfusion made no difference in allograft function 48 hours after transplant [53]. Further clinical trials are needed to determine the type, dose, and timing of antioxidant intervention.

### 7.3. Renin-Angiotensin System Blockade

Angiotensin II activates TGF-*β*1, apoptosis, oxidative stress, and atherogenesis in the cardiovascular system and the kidney [54–56]. Renin-angiotensin system (RAS) blockade has antifibrotic and antiproteinuric properties in experimental and clinical studies of kidney diseases. Angiotensin II, acting via aldosterone, increases plasminogen activator inhibitor-1 (PAI-1) and limits plasmin production from plasminogen, resulting matrix overproduction. Angiotensin-converting enzyme inhibitors (ACE-I) and angiotensin (Ang) II receptor blockade lead to reduced intragraft expression of TGF-*β* and reduced proteinuria. Therefore, this has now become the first line therapy in patients with CKD and hypertension or proteinuria [57].

A large retrospective study of over 2000 RT recipients showed that 10-year patient and graft survival was significantly improved in individuals treated with ACE-I or Ang II receptor blockers [58]. A meta-analysis of 21 randomised controlled trials including 1549 patients showed that RAS blockade was associated with significant drop in haematocrit (−3.5%), GFR (−5.8%), and proteinuria (−0.47 g/d) during a 2-year time period, but there are insufficient data to determine the effect on patient or graft survival [59].

In comparison with patients with native kidney disease, there is little information from prospective randomised controlled trials examining the effects of RAS blockade on long-term outcomes in RT. The ongoing Canadian ACE-I trial (ISR-78129473) and American Angiotensin II Blockade for the prevention of cortical interstitial expansion and graft loss in RT recipients (NCT00067990) studies will provide clinically meaningful evidence on the effect of RAS blockade on patient and graft survival in RT recipients [59, 60].

### 7.4. Inhibition of TGF-*β*1

TGF-*β*1 is upregulated in animal and human allografts undergoing chronic rejection and in chronic cyclosporine-induced tubulointerstitial fibrosis [61, 62]. On the other hand, TGF-*β*1 exerts immunosuppressive effects in the RT and helps the generation of T-regulatory cells thereby inducing certain degree of graft tolerance, which is beneficial to the graft. Therefore, caution needs to be exercised in blockade of TGF-*β*1. For example, TGF-*β*1 knockout mice die at an early stage from uncontrolled multifocal inflammatory disease and evidence suggests that overexpression of TGF-*β*1 in early acute rejection may prevent chronic rejection and improved outcomes [63, 64]; hence, TGF-*β*1 may be a less than optimal target in transplant settings. Alternatively,* downstream targets* may be more useful approach. To date, no clinical trial has been undertaken on the use of TGF-*β*1 in human RT. Agents, used to inhibit TGF-*β*1, are described below.

#### 7.4.1. Pirfenidone

Pirfenidone (PFD) (5-methyl-a-phenyl-2-(1H)-pyridone) is an orally active synthetic agent that inhibits expression of TGF-*β*1, epidermal growth factor, PDGF, and fibroblast proliferation [65]. In a rat model of cyclosporine nephrotoxicity, PFD reduced TGF-*β* mRNA protein expression and fibrosis by 50% and reduced proapoptotic gene expression [65, 66]. Administration of PFD in focal segmental glomerulosclerosis led to 25% improvement in the rate of decline of kidney function [67]. PFD has not been tested in human clinical transplantation yet.

#### 7.4.2. Relaxin

Relaxin is a peptide hormone, a member of insulin growth factor (IGF) family, and a naturally occurring inhibitor of collagen deposition during normal development, aging, and pregnancy. In cultured human renal fibroblasts, exposure to relaxin inhibited TGF-*β*-induced type I collagen and fibronectin synthesis and signalling via SMAD2 and also stimulated matrix metalloproteinase (MMP)-2 and -9 secretion [68]. Furthermore, in a relaxin gene-knockout mouse, the progressive renal fibrosis and deteriorating renal function were reversed with recombinant relaxin [69]. In a bromoethylamine-induced model of renal fibrosis in the rat, relaxin administration was associated with a significant decrease in interstitial fibrosis at the corticomedullary junction, accompanied by a decrease in the number of ED-1 positive cells (an index of macrophage infiltration) and in the intensity of immunohistochemical staining for transforming growth factor-beta [70]. However, there have been no preclinical studies in transplantation.

#### 7.4.3. Decorin

Decorin is a small leucine rich proteoglycan that forms complexes with TGF-*β* leading to inhibition or sequestration within the ECM. In experimental models of obstructive uropathy, treatment with decorin reduced proteinuria, collagen deposition, and expression of TGF-*β* within the kidney [71, 72].

#### 7.4.4. Endothelin-1 Inhibition

Endothelin-1 (ET-1) is a vasoactive peptide with potent vasoconstrictive properties, which is produced in tubular epithelium, macrophages, and fibroblasts. ET-1 also promotes fibrogenesis by upregulating TGF-*β*, directly stimulates collagen synthesis, and limits collagen degradation [73]. The use of nonselective ET-1 receptor blockade in an ischaemic injury rat model limited the fall in GFR [74]. Bosentan, a nonselective ET antagonist, has been used in a rat tracheal allograft model where it ameliorated bronchiolitis obliterans, but the experience of ET-1 receptor blockade in clinical transplantation is limited [75].

#### 7.4.5. Bone Morphogenetic Protein-7 (BMP-7)

BMP-7 is a member of the TGF*β*-1 family, which signals through the ALK3 and ALK6 type I receptors to phosphorylate SMAD1, SMAD5, and SMAD8, whereas TGF*β*1 signals through the ALK5 type 1 receptor to phosphorylate SMAD2 and SMAD3 [76]. BMP-7 can counterbalance the profibrotic effects of TGF*β*-1 by the activation of regulatory SMAD1, SMAD5, and SMAD8. It was observed that renal allografts with tubulointerstitial fibrosis and EMT were associated with upregulation of intraepithelial phospho-SMAD2/3 and concomitant downregulation of phospho-SMAD1/5/8, whereas BMP-7 increased phosphor-SMAD1/5/8 in renal cortical epithelial cells in vitro [37].

#### 7.4.6. Connective Tissue Growth Factor (CTGF)

CTGF is heparin binding cysteine-rich protein that gets activated through SMAD and MEK pathways; thereby it activates TGF-*β*1 and inhibits the antifibrotic effects of BMP-7. The net effect leads to cellular proliferation, collagen synthesis, chemotaxis, and EMT [77, 78].

CTGF mRNA and protein levels were increased in a mouse model of kidney transplantation. In vitro studies showed CTGF to induce EMT in tubular epithelial cells. Furthermore, urinary CTGF levels were increased in RT recipients with chronic allograft fibrosis [79]. In Fisher-to-Lewis RT model, CTGF silencing with siRNA decreased allograft fibrosis [80]. No studies on the role of CTGF in clinical RT have been performed.

#### 7.4.7. SMAD and Rho GTPases Inhibition

TGF-*β*1 activates SMAD 2/3 by phosphorylation through the ALK type I receptor. It can also activate Ras/rho and the downstream MEK pathway through noncanonical SMAD-independent pathway [81]. Targeting these downstream molecules in the TGF-*β*1 signalling pathway is an alternative antifibrotic strategy.

In experimental models, inhibition of SMAD3 and rho prevented fibrogenesis. In unilateral ureteric obstruction (UUO) model of SMAD3 knockout mice, minimal renal fibrosis was observed [82]. In the UUO mice model, a rho-associated coiled-coli forming protein kinase inhibitor (Y-27632) prevented the transcription of fibrosis genes including TGF-*β*1, *α*-smooth muscle actin (*α*-SMA), and *α*1-collagen and matrix deposition [83]. Fasudil, a specific rho kinase inhibitor, attenuated myocardial fibrosis and interstitial fibrosis in experimental models of diabetic and obstructive uropathy [84]. However, no preclinical or clinical studies have examined the role of such intervention in renal allografts.

#### 7.4.8. Vascular Endothelial Growth Factor (VEGF)

VEGF is an antigenic factor expressed in glomerular podocytes and distal tubules in response to stimuli such as hypoxia, TGF-*β*1, epidermal growth factor, and PDGF. VEGF is an inducer of proliferation (extracellular signal-regulated kinases), permeability (endothelial fenestration), invasion (matrix metalloproteinases), and survival (activation of Akt/P13K, caspase inhibition) [85].

One study has examined the influence of VEGF on renal function and development of interstitial fibrosis in renal allografts in 92 patients with acute rejection, CAN, and acute cyclosporine toxicity. Increased VEGF expression was correlated with increased expression of TNF-*α* levels and macrophage infiltration, associated with increased risk of early interstitial fibrosis and poor graft outcome in long term [86].

#### 7.4.9. Hepatocyte Growth Factor (HGF)

Hepatocyte growth factor (HGF) was originally identified and cloned as a potent mutagen for mature hepatocytes. It is now clear that HGF acts on various types of cells through its MET receptor tissue kinase and elicits pleiotropic effects involved in embryogenesis and tissue repair [87].

HGF prevents the initiation and progression of renal fibrosis by inhibiting TGF-*β*1 expression, myofibroblasts activation, and EMT. It can block the nuclear translocation of SMAD-2/3 and upregulate the expression of SMAD transcriptional compressors, Sloan Kettering Institute (SKI)-related novel protein N (SnoN), and TG-interacting factors (TGIF) [88].

In a rat model of CAN, treatment with human recombinant HGF prevented renal allograft inflammation (decreased TNF-*α*, MCP-1, and iNOS mRNA levels) and fibrosis (decreased TGF-*β*1 mRNA and matrix accumulation) [89]. Similar results were observed following human HGF gene therapy immediately before and after RT in rats [90]. However, HGF therapy in RT recipients may be associated with the potential risk of cancer, given that the HGF receptor is a tyrosine kinase receptor involved in progression of carcinomas and metastasis [91].

### 7.5. Inhibition of Matrix Deposition

#### 7.5.1. Prolyl-4-hydroxylase Inhibitors

In collagen synthesis, prolyl-4-hydroxylase is essential in posttranslational modification of the alpha chains of procollagen. Inhibition of this enzyme prevents hydroxylation of proline of the procollagen chain, subsequently causing intracellular degradation of the procollagen and reduction in the collagen deposition in the interstitium [92].

Phenanthrolinone, a competitive inhibitor of prolyl-4-hydroxylase, was studied in a murine model of CAN, which demonstrated reduction in inflammation and graft fibrosis along with improvement in graft function. The drug was used once wounds had healed. Clinical application of this agent needs to be established [93].

#### 7.5.2. MMP-2, MMP-3, and MMP-9 Inhibitor (Bay 12-9566)

In rat renal allografts, fibrosis is associated with increased expression of MMP-2 and MMP-9 and decreased tissue inhibitor of matrix metalloproteinases (TIMP)-3 [94]. BAY 12-9566, an inhibitor of MMP-2, MMP-3, and MMP-9 given early in the posttransplant period, improved proteinuria and histology. However, institution at late time-point appeared to aggravate disease [95].

#### 7.5.3. Retinoids

Retinoids have been recognised for their anti-inflammatory capacity and their specific receptors are expressed within the kidney as well as T- and B-cells and macrophages [96]. Treatment with isotretinoin (13-cisRA) in a chronic rat allograft model reduced interstitial fibrosis and inflammatory cell infiltration and is considered as an important therapeutic approach when chronic rejection and immune response are implicated in the allograft injury [97].

### 7.6. Platelet-Derived Growth Factor (PDGF) Inhibitors

PDGF is a family composed of PDGF-A, PDGF-B, PDGF-C, and PDGF-D, which are potent growth factors for myofibroblasts, and exerts their cellular effects by binding to tyrosine kinase receptors-*α* and -*β*. PDGFs play pivotal roles in wound healing, regulation of interstitial fluid pressure, malignancies, atherosclerosis, and fibrotic diseases [98]. The increased expression of PDGF has been observed in both animal (mesangioproliferative and rat transplant models) and human (proliferative GN and diabetic nephropathy, transplant glomerulopathy) renal diseases [99–102]. PDGF was present at glomeruli and proximal tubular cells and in areas of peritubular, interstitial, and periglomerular fibrosis.

In the Dark Agouti to Wistar-Furth rat model of CAN, imatinib (STI571), a selective PDGF receptor tyrosine kinase inhibitor, prevented the development of CAN and preserved renal function [103]. In the same model, similar results were observed with FK778, a synthetic analogue of an active metabolite of leflunomide, which inhibited de novo biosynthesis of pyrimidine and prevented activation of both T- and B-lymphocytes and expression of TGF-*β* ligand and receptor [104].

### 7.7. Nuclear Factor Kappa-B (NF-*κ*B) Signalling Inhibitors

NF-*κ*B comprises a family of transcription factors. NF-*κ*B pathway is activated by TNF-*α*, IL-1, and LPS or stress-mediated cascades. Upon translocation of NF-*κ*B into the nucleus from cytoplasm, it binds to the DNA and regulates production of various cytokines, chemokines, stress response proteins, and antiapoptotic proteins.

Activation of NF-*κ*B is observed in both animal (rat model of glomerulonephritis) and human (diabetic nephropathy) renal diseases [105, 106]. The NF-*κ*B positive nuclei were seen in the mesangial cells, endothelial cells, podocytes, tubular cells, and mononuclear infiltrates in the interstitium. In both studies, phosphorylation of p38MAP kinase was observed.

Inhibition of NF-*κ*B using pyrrolidone dithiocarbamate (PDTC), steroids (prednisolone and dexamethasone), gliotoxin, parthenolide, and proteasome inhibitor N-benzyloxy-carbonyl-Ile-Glu (o-t-Bu)-Ala-Leucinal has been examined in rat models. Significant reduction in inflammation, development of fibrosis by reducing MCP-1 gene, and profibrotic gene expression were observed [107–109].

Activation of NF-*κ*B was inhibited on human proximal tubular cells when treated with mycophenolic acid [110]. Bortezomib, a proteasome inhibitor, was shown to reduce antibody production and reduce vasculopathy in rat cardiac transplantation models [111]. Case reports on the use of Bortezomib for antibody-mediated rejection are available in human renal transplantation, but its clinical application is still in experimental stage [112].

## 8. Biomarkers of CAN 

Several biomarkers of CAN have been examined for early detection and prediction of CAN, which still remain in investigative stage. Chemokine (C-C motif) ligand 2 (CCL2), also known as monocyte chemotactic protein-1 (MCP-1), recruits monocytes, memory T cells, and dendritic cells to the sites of tissue injury, infections, and inflammation. Urinary CCL2 was measured and protocol biopsies performed prospectively in 111 RT recipients at 0, 6, and 24 months, which demonstrated urinary CCL2 at 6 months as an independent risk factor for subsequent development of IFTA at 24 months, both in univariate and multivariate analyses [113].

Proteomic analysis of blood samples using mass spectrometry has identified several unique signatures of transcript and protein biomarkers with high predictive accuracies for mild and moderate/severe CAN, which can be used for proteogenomic classification of CAN based on peripheral blood profiling, although the validity remains to be proven [114, 115].

In 2003, Scherer et al., in their genomics study using microarray technology, detected upregulation of several genes, which could predict the development of CAN. Those genes were APRIL (acidic protein rich in leucines), OBCML (opiate-binding protein-cell adhesion molecule-like), the tumour suppressor gene NPRL2, cytokeratin 15, homeobox gene B7, prolactin receptor, and guanine nucleotide-binding protein g7 [116]. The same group also demonstrated early changes in several transcriptomes post-RT, which could predict development of CAN and identify patients at risk [117]. More recently, Einecke et al. examined RT biopsy specimens that showed genes associated with graft failure were related to tissue injury, epithelial dedifferentiation, matrix remodelling, and TGF-*β*. In multivariate analysis, molecular risk score, peritubular capillary basement membrane multilayering, arteriolar hyalinosis, and proteinuria were independent predictors of graft loss [118].

Oetting et al. from Minnesota have investigated the effect of telomere length (TL) on the allograft survival and CAN by measuring TL in DNA isolated from peripheral blood in 1805 recipients and 1038 living kidney donors using the multiplexed monochrome quantitative polymerase chain reaction assay. They concluded that the CAN was not associated with shorter TL, although older donor chronological age was associated with increased risk of CAN [119]. Molecular profiling is a newer advancement in identifying molecular signatures related to CAN. Maluf et al. have identified calcineurin inhibitor toxicity at the molecular level as a nonimmunological factor involved in the progression to CAD [120].

## 9. Conclusions

CAN, once established, is irreversible [3, 23]. Delaying the progression of renal fibrosis and preservation of allograft function should be the goal, which is being achieved through substitution with less nephrotoxic immunosuppressive agents and modification of risk factors, such as adequate control of hypertension, diabetes, hyperlipidaemia, proteinuria (angiotensin blockade), and infections (CMV, BKV, and urine tract infections). CNI minimisation and steroid-sparing regimens were shown to reduce the progression of CAN [121, 122]. Substitution of CNIs with sirolimus and mycophenolate mofetil leads to improvement and preservation of renal function in CAN cases [123, 124]. Early diagnosis of CAN through protocol biopsies and institution of appropriate immunosuppressive regimens and treatment of subclinical rejection is essential to prevent late diagnosis of CAN [125].

Several interventional strategies have been examined to block the intracellular and extracellular cascades of events at molecular levels in both clinical and experimental settings to prevent CAN, but limited success has been achieved [33, 49]. Preventive and treatment strategies targeting TGF-*β*1 signalling pathway are reasonable antifibrotic options in RT, but TGF-*β* expression in RT is being considered to be beneficial because of its effect in gaining tolerance [33]. More specifically, PFD and therapies targeting BMP-7, HGF, and CTGF, although having shown promising results, still are in the experimental phase [76]. Exploration of alternative pathways and downstream molecules is critical for developing new strategies to ameliorate graft fibrosis and atrophy. Clinical trials are needed to examine their long-term effects in RT. Modulation of the risk factors, both immunological and nonimmunological, have been successful in slowing down the progression of CAN to some extent, but not successful in prevention or reversal of the ultimate changes of CAN [126–128].

## Figures and Tables

**Table 1:** Risk factors implicated in CAN.

Immunological risk factors	Nonimmunological risk factors
Histocompatibility Acute rejection episodes Suboptimal immunosuppression Subclinical rejections Anti-donor antibodies Noncompliance	Ischaemia-reperfusion injury Brain death Infection (cytomegalovirus and BK virus) CNI toxicity Donor factors: age, hypertension, smoking, diabetes, gender, and reduced renal mass Recipient factors: race, hypertension, smoking, diabetes, and hyperlipidaemia
